# Ocular blood flow and choroidal thickness changes after carotid artery stenting

**DOI:** 10.5935/0004-2749.20200081

**Published:** 2024-02-11

**Authors:** Esra Biberoglu, Muhsin Eraslan, Ipek Midi, Feyyaz Baltacioglu, Macit Bitargil

**Affiliations:** 1 Department of Ophthalmology, Marmara University Pendik Educational and Research Hospital, Istanbul, Turkey; 2 Department of Neurology,Marmara University Pendik Educational and Research Hospital, Istanbul, Turkey; 3 Department of Radiology, Marmara University Pendik Educational and Research Hospital, Istanbul, Turkey; 4 Sisli Etfal Hamidiye Educational and Research Hospital, Istanbul, Turkey

**Keywords:** Carotid stenosis, Stents, Ultrasonography, doppler, color, Choroid/anatomy & histology, Ciliary arteries, Estenose das car**ó**tidas, Stents, Ultrassonografia doppler em cores, Coroide/anatomia & histologia, Art**é**rias ciliares

## Abstract

**Purposes:**

To evaluate changes in ocular blood flow and subfoveal choroidal thickness in patients with symptomatic carotid artery stenosis after carotid artery stenting.

**Methods:**

We included 15 men (mean age, 63.6 ± 9.1 years) with symptomatic carotid artery stenosis and 18 healthy volunteers (all men; mean age, 63.7 ± 5.3 years). All participants underwent detailed ophthalmologic examinations including choroidal thickness measurement using enhanced depth-imaging optic coherence tomography. The patients also underwent posterior ciliary artery blood flow measurements using color Doppler ultrasonography before and after carotid artery stenting.

**Results:**

Patients lacked ocular ischemic symptoms. Their peak systolic and end-diastolic velocities increased to 10.1 ± 13.1 (p=0.005) and 3.9 ± 6.3 (p=0.064) cm/s, respectively, after the procedure. Subfoveal choroidal thicknesses were significantly thinner in patients with carotid artery stenosis than those in the healthy controls (p=0.01). But during the first week post-procedure, the subfoveal choroidal thicknesses increased significantly (p=0.04). The peak systolic velocities of the posterior ciliary arteries increased significantly after carotid artery stenting (p=0.005). We found a significant negative correlation between the mean increase in peak systolic velocity values after treatment and the mean preprocedural subfoveal choroidal thickness in the study group (p=0.025, r=-0.617).

**Conclusions:**

In patients with carotid artery stenosis, the subfoveal choroid is thinner than that in healthy controls. The subfoveal choroidal thickness increases after carotid artery stenting. Carotid artery stenting treatment increases the blood flow to the posterior ciliary artery, and the preprocedural subfoveal choroidal thickness may be a good predictor of the postprocedural peak systolic velocity of the posterior ciliary artery.

## Abstract (pt)

**Objetivos:**

Avaliar alterações no fluxo sanguíneo ocular e na espessura da coroide subfoveal em pacientes com estenose sintomática da artéria carótida, após implante de stent nessa artéria.

**Métodos:**

Foram incluídos 15 homens (idade média de 63,6 ± 9,1 anos) com estenose sintomática da artéria carótida e 18 voluntários saudáveis (todos homens; idade média de 63,7 ± 5,3 anos). Todos os participantes foram submetidos a exames oftalmológicos detalhados, incluindo d medição da espessura da coroide, usando tomografia de coerência óptica com imagem de profundidade aprimorada. Os pacientes também foram submetidos a medidas do fluxo sanguíneo das artérias ciliares posteriores, usando ultrassonografia com Doppler colorido, antes e após o implante do stent na artéria carótida.

**Resultados:**

Os pacientes não apresentaram sintomas isquêmicos oculares. O pico de velocidade sistólica e diastólica final aumentou para 10,1 ± 13,1 (p=0,005) e 3,9 ± 6,3 (p=0,064) cm/s, respectivamente, após o procedimento. As espessuras da coroide subfoveais foram significativamente mais finas nos pacientes com estenose da artéria carótida do que nos controles saudáveis (p=0,01). Porém, durante a primeira semana pós-procedimento, as espessuras das coroides subfoveais aumentaram significativamente (p=0,04). O pico de velocidade sistólica das artérias ciliares posteriores aumentou significativamente após o stent na artéria carótida (p=0,005). Encontramos uma correlação negativa significativa entre o aumento médio dos valores máximos de velocidade sistólica após o tratamento e a espessura da coroide subfoveal pré-procedimento média no grupo de estudo (p=0,025, r=-0,617).

**Conclusões:**

Em pacientes com estenose da artéria carótida, a coroide subfoveal é mais fina que a dos controles saudáveis. A espessura da coroide subfoveal aumenta após o stent na artéria carótida. O tratamento com stent na artéria carótida aumenta o fluxo sanguíneo para a artéria ciliar posterior, e a espessura coroidal subfoveal pré-procedimento pode ser um bom preditor da velocidade sistólica de pico pós-procedimento da artéria ciliar posterior.

## INTRODUCTION

Early ophthalmologic examinations may help prevent irreversible disorders and blindness. They may also hint to carotid artery stenosis (CS) and help prevent complications such as stroke^(1)^. The main purpose in the treatment of CS is to eliminate the stenosis and its related strokes^(2)^. Improvements in ocular blood flow after carotid artery stenting (CAS) may be demonstrated using color Doppler ultrasound (CDUS)^(3)^. Studies have shown choroidal thickness being reduced in patients with CS^(4)^. Our aims with this study were to determine ocular blood flow changes in patients before and after CAS, to grade the improvement in retinal and choroidal blood flows by recording subfoveal choroidal thicknesses (SFCTs) using optical coherence tomography (OCT), and to compare the blood flow improvements with ciliary artery blood flow changes using CDUS.

## METHODS

We diagnosed 15 patients (all men; mean age, 63.6 ± 9.1 years) with transient ischemic attack or stroke as having >70% CS using CDUS or carotid magnetic resonance (MR) angiography and treated them with CAS in our interventional radiology unit. We included all those patients and 18 healthy volunteers (all men; mean age, 63.7 ± 5.3 years) for the control group into our study.

We recorded the participants’ clinical history details focusing on hypertension, diabetes, hyperlipidemia, alcohol consumption, and smoking. The patients underwent ophthalmologic examinations before stenting and during the first week, first month, and third month afterwards. We also performed detailed eye examinations including slit lamp anterior segment examinations on all controls. We assessed best-corrected visual acuities of all subjects using Snellen visual acuity (VA) charts. We converted Snellen VA scores to LogMAR values for statistical comparison.

**Figure 1:**
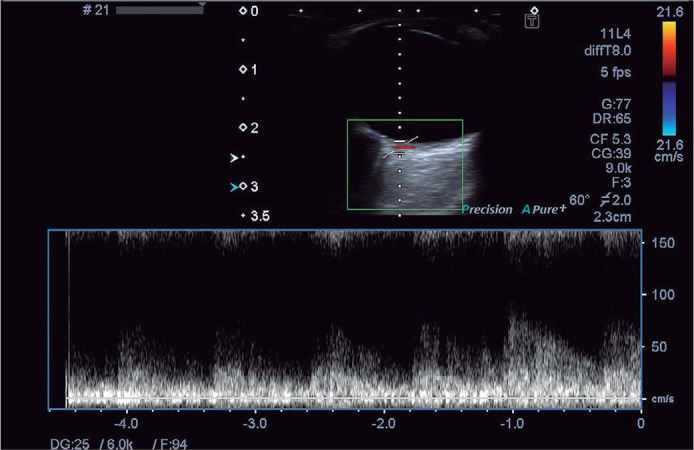
Doppler ultrasound images of posterior ciliary artery.

We scheduled orbital CDUS examinations for patients included in the study and receiving CS treatment and for the healthy volunteers. We performed all posterior ciliary artery (PCA)-oriented analyzes using a Toshiba Aplio 500 with a 6 MHz high-frequency linear array transducer. We examined the patient group using CDUS immediately before and after the treatment. The same radiologist examined all participants (in the supine position with their eyes closed) using the same device. All Doppler imaging procedures were performed between 11.00 am and 13.00 pm to avoid diurnal variations in ocular blood flow. The radiologist applied conductive gel on the closed eyelids and was careful not to apply too much pressure on the globe. Horizontal and vertical gray-scale scanning images of the orbit helped exclude possible pathologies. The radiologist localized the PCAs considering the orbital anatomy and measured the blood flow velocities by setting the appropriate marker angles to the vessel tract. The radiologist also measured and recorded peak systolic velocity (PSV), end-diastolic velocity (EDV), and resistivity index (RI) values (Figure 1).

**Figure 2:**
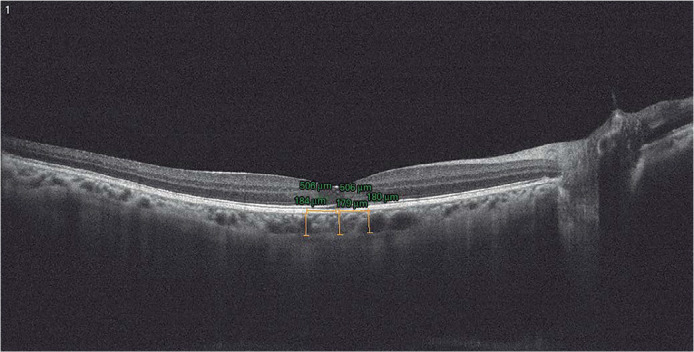
Subfoveal choroidal thickness measurements by EDI-optical coherence tomography.

We assessed all participants using the RTVue RT100 Spectral Domain OCT (Optovue, Foremont, CA, USA) device. We selected the line mode for choroidal imaging for choroidal assessment and took 12 mm cross-sectional images passing through the optic nerve and fovea center. We repeated choroid imaging twice for each eye. We measured systemic tension arterial pressures before each OCT exam. We took SFCT measurements on three sites: at the center of the fovea, on the 500-micron temporal portion of the center of the fovea, and on the 500-micron nasal portion. We performed SFCT measurements manually by measuring the distance between the outer edge of the hyper-reflective band of the retinal pigment epithelium and the inner boundary of the scleral hyper-reflectivity. All OCT scans were performed between 11.00 am and 13.00 pm to avoid choroidal thickness diurnal variations. In addition, for the analysis of SFCTs, we compared the average SFCT age distributions of the patients with those of 18 ageand gender-matched healthy controls. The same experienced physician performed all OCT scans and SFCT measurements and recorded the averages of the two measured SFCT values (Figure 2).

The same ophthalmologist also measured the central corneal thicknesses (CCT) and the axial lengths of all subjects using a Haag-Streit International/LS 900 Lenstar. We dilated the patients’ pupils with tropicamide and phenylephrine eye drops to assess lens and cataract statuses. We then performed detailed fundus examinations including the entire retinal periphery using a Volk SuperField NC lens making sure to investigate venous stasis retinopathy, iris neovascularization, glaucoma, optic nerve injury, vascular emboli, occlusion, and ocular ischemic syndrome (OIS).

We excluded patients with a VA lower than 6/10; those with refractive errors higher than -4 and +3D spherical and ≤ ± 3D cylindrical; those with uveitis, glaucoma, or retinal diseases; those with optic disk damages and corneal and vitreal opacities; those who had undergone ocular surgery except phacoemulsification; those with cataract (NC<4, C<5, P<3 according to LOCSS III classification); and those using medications for systemic diseases that might affect the ocular measurements.

The institutional review board and ethics committee of Marmara University approved the protocol of the study, and we obtained written informed consents from each participant prior to the examinations.

### CAS procedure

Before the CAS procedure, we provided individuals with detailed information about the treatment and its possible complications. We initiated patients on dual anti-aggregant therapy (75 mg clopidogrel + 100 mg ace tylsalicylic acid per day) 7 days before the stent procedure. We treated patients who had not received dual anti-agregant treatment and needed urgent interventions with 450 mg clopidogrel loading doses. All patients underwent treatment in an angiography unit equipped with a Siemens Artis Zee Bi-plain angiography device. At the beginning of the procedure, all patients were given 5000 U heparin intravenously. We treated all patients with CAS and a dual filter protection with the Boston Scientific Filter Wire EZ Embolic Protection System in 13 patients and the Spider FX™ Embolic Protection Device in 2 patients. We used Cristallo Ideale™ Carotid Stent System Self-Expanding stents in 11 patients and Protege^®^ RX Carotid Stent System Self-Expanding Nitinol stents in 4 patients. We performed pre-dilatation with 3 × 20 mm balloons in four patients before stenting to safely pass through the lesion due to their high stenosis grades. We also performed post-dilations to achieve optimal stent openings after opening all stents; we used 6 × 20 mm balloons in six patients and 5 × 20 mm balloons in nine patients. After completing the procedures, we scheduled follow-up angiograms to evaluate the status of the neck and intracranial arteries.

During the postprocedural period, we prescribed dual anti-aggregant therapy (75 mg/day clopidogrel + 100 mg acetylsalicylic acid) for 3 months, followed by lifelong 100 mg acetylsalicylic acid or clopidogrel prophylaxis according to the drug resistance results.

### Statistical analysis

We used the Statistical Package for the Social Sciences for Windows version 21.0 to perform all statistical analyzes. We expressed descriptive statistics with means ± standard deviations and percentage values. We assessed the conformity of the data to normal distribution using the Kolmogorov-Smirnov test, and we used parametric tests to analyze numeric data with normal distributions and non-parametric tests to analyze numeric data with non-normal distributions.

We applied Student’s T test and the Mann-Whitney U test for intergroup comparisons and the paired intragroup comparisons of means. We applied the Chi-square test for the analysis of proportional data. We performed correlation analyzes using the Pearson test and the Wilcoxon test for the analysis of repeated samples. For p values Friedman test we used for compared three or more matched or paired groups. We accepted statistical significance levels at p<0.05. For analyzing variables from the same family together, we decided the significance level according to Bonferroni corrections based on p<0.05.

## RESULTS

We found no significant differences between the patients with CS and the controls in terms of mean age (p=0.913), spherical equivalent (p=0.986), VA (p=0.343), CCT (p = 0.376), or axial length (p = 0.065). No patients had eye pain, retinal hemorrhage, or glaucoma. No patients had complications following stenting.

We found a statistically significant difference between the controls and the patients in terms of the CS of SFCT values. These results suggest a decrease in the SFCTs of patients with CS compared with the SFCTs of the individuals in a normal population (Table 1). The postprocedural SFCT measurements increased. We found a significant difference between the preprocedural SFCT and the postprocedural first week measurement (p=0.04) (Table 2).

**Table 1:** Comparison of SFCT values between the control and study groups

		Control group	Study group	p-value
SFCT	Preprocedural	302.05 ± 62.09	216.09 ± 52.00	0.01^*^
	Postprocedural 1^st^ week	302.05 ± 62.09	226.90 ± 61.40	0.005^*^
	Postprocedural 1^st^ month	302.05 ± 62.09	240.16 ± 60.30	0.023^*^
	Postprocedural 3^rd^ month	302.05 ± 62.09	234.64 ± 58.67	0.012^*^

SFCT= subfoveal choroidal thickness.

Values are means ± standard deviations unless otherwise indicated.

* p<0.05, Student’s t test.

**Table 2:** Comparison of SFCT values within the study group

	Preprocedural	Postprocedural 1^st^ week	Postprocedural 1^st^ month	Postprocedural 3^rd^ month	p-value
SFCT (µm)	216.09 ± 52.00	226.90 ± 61.40	240.16 ± 60.30	234.64 ± 58.67	0.118^*^
p-value		0.004^**^	0.123^**^	0.916^**^	

SFCT, subfoveal choroidal thickness.

Values are expressed as means ± standard deviations unless otherwise indicated.

* p<0.05 Friedman was used for p values.^**^Based on Wilcoxon test.

The mean preprocedural PSV and EDV values of the patients in the study group after treatment increased by 10.1 ± 13.1 and 3.9 ± 6.3 cm/s, respectively. The PCV increase was statistically significant (p=0.05). Although postprocedural RI values showed a decrease of 0.20 ± 0.18 units compared with preprocedural values, this was not statistically significant. Table 3 summarizes the comparison of preprocedural and postprocedural CDUS findings in the study group.

**Table 3:** Comparison of Doppler ultrasound findings between the control and the study groups and comparison of Doppler ultrasound findings within the study group (n = 13)

	Control group (n=16)^(a)^	Study group (n = 13)^(b)^		p-value		
		Preprocedural	Postprocedural	Control-pre procedural p-value	Control-post procedural p-value	Interstudy group pre-post comparison
PSV (cm/sec)	24.9 ± 23.8	19.8 ± 10.8	29.9 ± 15.7	0.714	0.075	0.005^*^
EDV (cm/sec)	7.9 ± 9.1	4.7 ± 2.8	8.6 ± 5.2	0.619	0.132	0.064
RI	0.68 ± 0.17	0.71 ± 0.15	0.69 ± 0.09	0.559	0.682	0.814

PSV= peak systolic velocity; EDV= end-diastolic velocity; RI= resistive index.

Values are mean ± standard deviation unless otherwise indicated.

* p<0.05 Friedman was used for p values.

We used Mann-Whitney U tests for comparisons between groups.

(a) We performed Doppler measurements in 16 participants of the control group;

(b) We performed Doppler measurements in 13 patients of the study group.

We found no statistically significant differences after comparing the preprocedural and postprocedural PSV, EDV, and RI values of PCA with those of the controls. Table 3 summarizes the CDUS findings and the comparison of p values between the control and the study groups.

We found highly significant positive correlations between the preprocedural PSV and the postprocedural PSV values (p=0.044; r=0.565), between the preprocedural PSV and the postprocedural EDV values (p=0.005; r=0.723), and between the postprocedural PSV and the postprocedural EDV values (p=0.003; r=0.746).

Also, we found a significant negative correlation between the increase in PSV values after treatment and the preprocedural SFCT in the study group (p=0.025, r=-0.617). The thickness of preprocedural SFCT was inversely proportional to the increase in PSV after CAS.

In the study group, we observed positive correlations between the preprocedural EDV values and the SFCT values on the first week and third month but not on the first month. We found no associations between other Doppler parameters and the SFCT values. Table 4 summarizes the association between preprocedural EDV and SFCT values in the study group.

**Table 4:** Association between preprocedural EDV and SFCT values in the study group

Preproceclural		SFCT			
		Postprocedural 1^st^ week	Postprocedural 1^st^ month	Postprocedural 3^rd^ month	p-value
Preprocedural		0.038^*^	0.045^*^	0.259	0.031^*^
EDV	r	0.579	0.586	0.495	0.754

SCFT= subfoveal choroidal thickness; EDV= end-diastolic velocity.

* p<0.05 Pearson’s correlation test.

As expected, we found a negative correlation between the age and the SFCT in the control group (p=0.025, r=-0.527).

## DISCUSSION

Carotid arteries are the main vessels feeding the brain and eyes. CS is an important disease that causes mortality and morbidity^(5)^. Atherosclerosis is the most important cause of the stenosis. CDUS, CT angiography, and MR angiography are all used for diagnosing CS^(6)^. CDUS is frequently preferred because of its lack of radiation, its noninvasive nature, and its cost-effectivity^(7,8)^. Stenting is an alternative to endarterectomy for the treatment of CS^(9)^.

Ocular involvement can be observed in internal carotid artery (ICA) stenosis, and ocular symptoms may be the first sign of carotid disease^(10)^. CS may cause chronic ocular ischemia^(11)^. The most common ischemic ocular symptom is acute transient monocular blindness due to an embolism in the retinal artery^(10)^. Ocular involvement may manifest itself with a wide range of symptoms including temporary acute unilateral blindness due to embolism released from atherosclerotic plaque, chronic OIS due to permanent hypoperfusion, or full blindness due to central retinal artery (CRA) or ophthalmic artery (OA) occlusions. Anterior segment findings include conjunctival and episcleral injection, anterior chamber inflammation, and iris neovascularization in cases with anterior segment ischemia. If neovascularization is not treated, it may progress into neovascular glaucoma^(12)^. Less often, corneal edema and swollen cataracts may develop. Posterior segment signs of CS are more frequent than anterior segment signs^(13)^ and include narrowed retinal arteries, dilated retinal veins, patchy hemorrhages, microaneurysms, cotton wool spots, and neovascularization^(14)^. OIS is more common in patients with CS and poor collateral connections. The decrease in retrobulbar blood flow in patients with OIS can be demonstrated using CDUS. In some patients, a retrograde flow is seen as a predictive indicator of high-grade CS. This counter-current leads to further worsening of ocular ischemia by reducing retrobulbar blood flow with steal phenomenon^(15-17)^.

CS should be suspected when hemispheric neurologic symptoms, amaurosis fugax, and Hollenhorst plaques are seen. Retinal artery occlusion and ischemic optic neuropathy may also be associated with CS, as mentioned above^(18)^. In our study, no patients presented rubeozis iridis, neovascular glaucoma, or retinal artery or venous occlusions. Any stenoses of the ICA affect both retinal and choroidal blood flows.

Vascular blood flow is affected by two main factors: the first is the resistance inherent to the vessel wall, and the second is the pressure change between the ends of the vessel (blood vessel elasticity). Although the first agent is defined by cardiac function, the proportional location of the arterial vessel in the cardiac circulatory system depends on the main physiological state of the arterial vessel. RI is not affected by Doppler flow and can be applied to represent the elasticity and resistance of the downstream blood vessels. PSV is the highest spot over the length region of the spectrum, whereas EDV is the finish spot of the cardiac (heart) cycle^(19)^.

The choroid is a vascular structure consisting of cav ernous spaces. Stenting improves OA ocular blood flow^(20)^. The choroidal thickness can be measured using EDI-OCT, a noninvasive method for enhanced depth imaging^(21)^. The first study measuring choroidal thicknesses with OCT was performed in 2008 by Spaide et al. Others reported that the reasons for differences in the results between studies were disparities in OCT light sources and software used and differences between the demographic data of the study groups (ethnicity, mean age, refractive errors, and axial length)^(21-23)^.

The choroid plays an important role in the pathogenesis of choroidal ocular diseases. No publications have shown choroidal thickness reductions on the affected side in patients with OIS^(24)^. A study involving 19 patients with OIS and unilateral CS reported that the choroidal circulation decreased because of CS and accordingly the choroidal thickness and volume decreased^(25)^.

A study in normal subjects showed a negative correlation between short-term PCA RI (mean, 0.61 ± 0.07) and SFCT (mean, 319.9 µm ± 83.7 µm)^(26)^. The increase in the RI of the retrobulbar artery resulted in a decrea se in choroidal thickness. Therefore, low choroidal flow is associated with thinner choroidal thickness in healthy individuals^(26)^. Agladıoğlu et al found a negative correlation between choroidal thickness and the ICA RI (p=0.017) in a study comparing 43 patients with CS and 43 healthy individuals^(27)^.

In our study, the SFCT values of the control group showed a negative correlation with age, as noted before in the literature^(27)^. Also, both preand post-treatment SFCT values were significantly lower in the patient group than those in the control group. This suggests that in patients with CS, the blood flow to the OA and the PCA, which feeds almost the entire choroidal circulation, is diminished, and therefore, the SFCT is thinner than that in healthy individuals. This is true even with the slight increase in SFCT due to the increase in blood flow to the PCA after CAS after the first week, first month, and third month. Only the SFCT increases on the first week were statistically significant. The RI value (which shows the resistance) was higher in the patient group than that in the control group. We found a decrease of 0.2 units in the mean RI value after stenting in the patient group (as expected). However, this decrease was not statistically significant (RI decreased after treatment, and SFCT values increased). Our results suggest that when the pre-treatment SFCT is thin, a high PSV improvement may be expected after stenting. Preprocedural SFCT may be useful for predicting the increase in PSV of the PCA, but this should be confirmed by prospective randomized studies.

In patients with CS, ocular vessels usually have decreased systolic and diastolic blood flow velocities in CDUS, especially in OA and CRA currents. Decreased OA flows may have important effects on the circulation of the eyes, and collateral blood flow originating from the OA may provide important contributions to brain perfusion, even if not fully exploited^(3)^.

Some authors have mentioned that over 70% of CS, bilateral severe stenosis, and reverse current observation in OA may be risk factors for the development of OIS^(28-30)^. Geroulakos et al found a significant increase in post-treatment CDUS values and improvement in low VA and pre-orbital pain after carotid endarterectomy^(30)^.

In our study, we found a significant increase in the PCA PSV flow in patients after carotid stenting, consistent with the literature^(3)^. The higher the PSV before treatment, the greater was the increase in PSV after treat ment. The 0.2-unit decrease in RI after treatment was not statistically significant, and we attribute it to the fact that our patient number was low. Again, in accordance with the literature, although the PSV and EDV values were low, and the RI value was high in the patient group compared with the values in the control group, the differences were not statistically significant. This may be due to ocular blood flow being affected by the collateral flow caused by CS compensator mechanisms. We also think that the absence of a statistical correlation between Doppler measurements was due to the susceptibility of measurements to hypertension, hypotension, and Doppler angle effects. In the study group, EDV values before treatment, 1 week after treatment, and 3 months after treatment showed a positive correlation between themselves and no significant correlations between other Doppler parameters or SFCT values.

The limitations of our study include the low patient count, the inability to record the systemic pressure values that may affect the measurements during Doppler procedures, the possibility that the angle of the hand holding the probe during Doppler procedure was not the same at each measurement, the weakness of repeatability and reliability of EDV and PSV values dependent on the angle, and the possibility that the hemodynamic flow in the eye may be affected by the collaterals and autoregulation mechanisms.

Early recognition and treatment of patients with carotid occlusive disease may prevent more serious complications. Eye examinations are important in these patients because CS may present with ocular findings. Directing patients with ocular ischemic signs and symptoms to neurology prevents the risk of stroke. In our study, CS administration increased CS blood flow to the PCA, which feeds the choroid. We showed this by CDUS measurements, and we confirmed the SFCT increase using EDI-OCT. Thus, our results suggest that CAS treatment improves ocular hemodynamics in CS. The thinning of SFCT in the OCT may help predict atherosclerotic changes in the carotid artery.

Using CDUS, we showed that the PCA blood flow decreased in patients with CS and that ocular blood flow increased after CAS. The choroid feeds from the PCA so the SFCT was decreased, secondary to a decrease of ocular blood flow in patients with CS, and the difference was significant compared with that in the control individuals. Findings of reduced SFCT in OCT analyses may help confirm the presence of atherosclerotic stenosis in the carotid artery.
